# Sustainable healthy diets from the lens of behavioral science: an actionable definition for designing relevant individual behavior change interventions

**DOI:** 10.1016/j.joclim.2026.100693

**Published:** 2026-05-23

**Authors:** Ujué Fresán, Paquito Bernard, Vera Araújo-Soares, Simon J Lloyd, Guillaume Chevance

**Affiliations:** a ISGlobal, Barcelona, Spain; b Univ Rennes, Inserm, EHESP, Irset [(Institut de Recherche en Santé, Environnement et Travail)] - UMR_S 1085, Rennes, F-35000, France; c Center for Preventive Medicine and Digital Health, Medical Faculty Mannheim, Heidelberg University, Heidelberg, Germany; d Independent Researcher; e Institute of Agrifood Research and Technology (IRTA), Sustainability inBiosystems Research Program, Torre Marimon, Caldes de Montbui, Barcelona, 08140, Spain

**Keywords:** Meat reduction, Eating behaviors, Dietary sustainability, Nutritional education

## Abstract

Changes in the food system are key for attaining the Sustainable Development Goals. This viewpoint i) contends that, alongside structural changes to the food production and distribution systems, the next years are decisive to foster individual behavior change for sustainable healthy diets, especially in high-income countries; ii) provides a set of behavior change techniques that can contribute to the design of individual interventions aimed at that dietary change; iii) highlights the main weaknesses of previous eating behavior interventions and suggest how they may be overcome, notably by addressing potential negative spillovers and trade-offs; and iv) provides an actionable definition of sustainable healthy diets for designing behavior change interventions. This viewpoint offers a relevant starting point for the design of future interventions targeting individual behavioral change for sustainable healthy diets from a holistic and multi-disciplinary perspective.

## 1 Introduction

The way we produce and consume food carries high social and environmental costs. Despite producing more than enough calories to feed the global population, distribution is highly unequal [1]. More than two billion people are suffering some type of food insecurity, leading to undernutrition as well as overweight and obesity [2]. More than one-third-of the food produced is lost or wasted [3]. Three billion people cannot afford a healthy diet, with unhealthy diets being the leading cause of morbidity and mortality worldwide [2]. Additionally, there are countless cases of forced labor conditions and unfair salaries across the food system [4]. From a planetary health perspective, the food system is responsible of around one-third-of all greenhouse gas (GHG) emissions worldwide [5,6]. Its contribution to global warming is so important that it has been postulated that even if the GHG emissions from all non-food-related sectors were immediately stopped and net zero from now on, emissions from the food system solely would still preclude reaching the Paris Agreement goal of limiting global warming to 1.5 degrees Celsius [7]. Beyond climate change, the food system currently uses about 70% of freshwater withdrawals and is a major source of water eutrophication [8,9]. Forty percent of the habitable land on Earth is used for growing our food or to feed farmed animals [10]. About 73% of the world’s deforestation is related to the food system [11], being the leading cause of habitat degradation and biodiversity loss [12]. The consequences of this environmental degradation is already noticeable (e.g., extreme weather events are further compromising food security), affecting especially the most vulnerable areas [13]. If the current food production and consumption patterns continue as usual, the global impact on the environment of the food system will increase by 50–90% by 2050 in comparison to 2010 values [14]. Therefore, rapid, effective and combined structural, technological and individual changes are needed to achieve a more resilient, sustainable and fair food system within planetary boundaries [15,16].

This viewpoint focuses on the specific role of individual behavior changes towards sustainable healthy diets (i.e., those healthy diets with low environmental impact, in which foods are obtained from fair and ethical sources) [17,18], from the consumer's perspective, and under the lens of a multi-disciplinary collaboration between behavioral, socio-economic and climate scientists as well as nutritionists.

## 2 Promoting individual eating behavior change: why, for whom, and how?

### 2.1 Why promote individual eating behavior change

The question of individual behavior change versus systems change is a false dichotomy [16,[19], [20], [21]]. Lifestyle choices are enabled and constrained by the physical environment, political contexts and infrastructures, but at the same time individual behaviors can spread into, and ultimately shape, social and cultural norms in a bottom-up fashion, thus leading to political and structural changes [15,22,23]. Whether being generated in a top-down or bottom-up fashion, radical changes in current individuals’ dietary patterns towards healthy diets with low environmental impact are essential for achieving a food system that fits in planetary boundaries, and contribute to ending the global syndemic of malnutrition [16,24,25]. Such healthy diets with low environmental impact, beyond differences in preferences and traditions of each specific culture, are characterized by being nutritionally-balanced patterns, mainly (if not totally) based on whole plant-sourced foods [2,25]. Shifting current dietary patterns towards such diets has the potential of halving the pressure of the food system on climate change, and reducing by 6-22% other environmental impacts, such as water and land use, or the application of fertilizers [16]. Indeed, at the individual level, changing eating behavior is one of the most effective strategies that someone omnivorous can undertake to reduce their environmental footprint. Food consumption is a major contributor to the consumer's environmental footprint, especially in high-income countries (in the European Union, it accounts for approximately 42% of the average citizen's footprint), primarily due to the consumption of animal-sourced foods such as meat and dairy products [[26], [27], [28], [29]]. Transitioning to healthy diets with low environmental impact also would have major impacts on human health, avoiding about 11 million deaths per year and reducing premature mortality by almost 20% [25,30]. Additionally, if opting for foods from sustainable sources, not only considering environmental but also the socioeconomic dimension of food (e.g., working conditions, economic fairness, gender equality, etc.), consumers could significantly contribute to achieving a fairer food system, and contribute to the achievement of many of the Sustainable Development Goals (i.e., goals adopted by the United Nations in 2015 as a universal call to action to end poverty, protect the planet, and ensure that by 2030 all people enjoy peace and prosperity) [31].

Encouragingly, early signs of this transition already are noticeable and should now be encouraged. For instance, a report shows that almost 40% of people in the United States note that environmental sustainability has an impact on their decision to buy certain foods and beverages, and a similar percentage say that knowing that the workers who produce, distribute, or serve the food are treated in a fair and equitable way is important [32]. In Europe, a study conducted across 7 countries (Germany, Denmark, Switzerland, Austria, Portugal, France and Belgium) shows that the number of Europeans deliberately consuming meat less frequently is growing rapidly at 23%, with health being the primary motivator reported, followed by sustainability [33]. This highlights an ongoing, relatively new interest towards sustainable healthy diets in consumers. This transition should now be strongly encouraged to help people change their behaviors and progressively create new food-related social norms [34].

### 2.2 For whom should individual eating behavior change be promoted

To be fair and effective, most behavior change initiatives promoting sustainable healthy diets should be first directed to the higher emitter groups, usually the individuals with the higher incomes, between and within countries [15,35,36]. Between countries, mathematical modeling studies suggest that the general adoption of sustainable healthy diets in developed countries could be an effective strategy in reducing GHG emissions by 70–90% and the use of resources by 5–50%, while improving people´s health [35,37]. The general adoption of healthy diets in low-income countries, however, would require an increased use of natural resources due to the need to address existing combinations of often inefficient farming systems and widespread dietary insufficiency in terms of both quantity and quality [35]. Further, non-sustainable eating behaviors in high-income countries can have social, economic and environmental consequences in low-income countries, and these can exacerbate food insecurity and environmental degradation in those areas (e.g., the boom of quinoa demand from Global North countries has led to biodiversity loss in the producer countries, conflicts over land among peasants, and a reduced accessibility to this staple food by low-income families) [38].

Within high-income countries, individuals’ dietary environmental impact is higher among men [39,40], and among those with higher socioeconomic status and incomes [36,41]. Food insecurity and hunger are experienced among people with the lowest incomes even in high-income countries [42]. Thus, individuals from upper socioeconomic level in high income countries, frequently men, should be targeted first if we are to achieve significant reductions in diet-related environmental impact and a fair food system as soon as possible. Similar groups in rapidly industrialising countries, such as China, also are key targets for behavioral scientists if we want to prevent emerging economies from following the unsustainable pathways of high-income countries [43].

### 2.3 How should individual eating behavior change be promoted

Two main approaches have been applied for promoting eating behavior change: *i*) interventions inspired by behavioral economics using large- (e.g., taxing and subsidizing foods) and micro-environmental modifications (so-called "nudges") to alter peoples’ behaviors with little cognitive engagement (e.g., [44,45]); and *ii*) individual behavioral change interventions targeting people's capacity, opportunity and motivation in order to help individuals revaluate their behaviors and adopt relevant modifications (e.g.,[46,47]). The combination of both approaches is required to achieve scaled and long lasting change in eating behaviors [20]. Indeed, although reshaping food environments can be effective in changing eating behaviors [15,44,48], this set of approaches is likely to be more powerful if combined with individual measures aimed at educating, raising awareness and motivating individuals directly [20]. Individuals' behavior change interventions also can contribute to the acceptability of political, structural and environmental modifications and to limiting potential forms of psychological reactance [49].

Behavior change interventions targeting individuals are also effective in changing eating behaviors on their own (increasing the consumption of certain food groups, such as fruits and vegetables [[50], [51], [52], [53]], and reducing the intake of others, such as meat [47,[54], [55], [56]]). Those behavioral interventions are composed of several “active ingredients”, usually reported and labeled in the literature under the term “behavior change techniques (BCT)” [57,58]. These techniques describe the content of behavioral change interventions by naming each specific individual component forming the intervention, such as “providing information about the health consequences”, “goal setting”, or “self-monitoring”. These techniques can be delivered in various formats, including clinical settings (e.g., with a trained nutritionist), educational programs (e.g., school-based interventions), face-to-face sessions (individual or group), or through digital platforms. They share similar goals with well-established methods such as motivational interviewing or cognitive behavioral therapy and are often used to complement them or, in a research setting, to report what was done within a behavior change intervention.

One of the challenges in designing individual behavior change interventions is to use a good “cocktail” of BCTs. In theory, BCTs can be selected with the aim to target specific modifiable factors/predictors, also called mechanisms of actions, that can cause/predict the targeted behavior [59]. For example, if one assumed that emotions (e.g. eco-anxiety) are positively associated with the consumption of fruits and vegetables [60], specific BCTs could be selected to explicitly target those emotions and ultimately lead to changes to the consumption of fruits and vegetables. A second option that can help identify relevant BCTs is to empirically review the literature to identify which techniques are associated with interventional efficacy. Following this second option, and as part of this perspective, we performed a scoping review of systematic reviews and meta-analyses of eating behavior change interventions to identify key BCTs that can effectively contribute to the promotion of successful eating behavior changes. Key results from this scoping review are presented in the following section (please see additional methodological details and findings of this review at: https://osf.io/etb4s/files/osfstorage).

## 3 Effective behavior change techniques for achieving individual eating behavior change

In the last years, several systematic reviews or meta-analyses explicitly testing BCT effectiveness for changing eating behaviors [[50], [51], [52], [53],^61^,^62^], or identifying intervention features associated with changes in eating behaviors, have been published (see a description of these individual studies at https://osf.io/etb4s/files/osfstorage) [47,[54], [55], [56],^63^]. Table 1 presents the behavior change techniques identified according to different food groups and defined according to the BCT taxonomy (v1) [57]. Overall, we identified 16 techniques that were associated with eating behavior change across the different reviews and meta-analyses. Some BCTs, such as “goal setting” or “self-monitoring of the behavior”, were positively associated with efficacy of several interventions considering different food groups, such as increases in fruit and vegetable intake [51,52], decreases in meat consumption [47,55], and change in overall diet, including fat and energy intake [63]. On the other hand, other BCTs were associated with intervention efficacy for changing some specific behaviors but not others. For instance, “information about emotional consequences” (e.g., emphasising animal suffering) was positively associated with reductions in meat consumption [54,55], but negatively with the promotion of fruits and vegetables in one systematic review and meta-analysis [51]. Finally, some BCTs were only associated with one food group, such as “information about others’ approval” that can positively contribute to reduced meat consumption [54].

**Table 1:** Associations between behavior change techniques (BCT) and eating behaviors outcomes.

BCT		Definition	Eating behavior change			
			Improve overall diet	Increase fruit and vegetable intake	Reduce meat consumption	Decrease SSB* and increase water
1.1	Goal setting	Set or agree on a goal defined in terms of the behavior to be achieved	**+**	**+**	**+**	
1.2	Problem solving	Analyse, or prompt the person to analyse, factors influencing the behavior and generate or select strategies that include overcoming barriers and/or increasing facilitators	**+**	**+**		
1.4	Action planning	Prompt detailed planning of performance of the behavior (must include at least one of context, frequency, duration and intensity)	**+**		**+**	
2.2	Feedback on behavior	Monitor and provide informative or evaluative feedback on performance of the behavior		**mixed**		
2.3	Self-monitoring of behavior	Establish a method for the person to monitor and record the outcome(s) of their behavior as part of a behavior change strategy	**+**	**+**	**+**	
3.1	Social support (unspecified)	Advise on, arrange or provide social support (e.g. from friends, relatives, colleagues,’ buddies’ or staff) or non-contingent praise or reward for performance of the behavior		**+**		
4.1	Instruction on how to perform a behavior	Advise or agree on how to perform the behavior			**+**	**+**
4.2	Information about antecedents	Provide information about antecedents (e.g. social and environmental situations and events, emotions, cognitions) that reliably predict performance of the behavior	**+**			
5.1	Information about health consequences	Provide information (e.g. written, verbal, visual) about health consequences of performing the behavior	**+**	**–**	**+**	
5.3	Information about social and environmental consequences	Provide information (e.g. written, verbal, visual) about social and environmental consequences of performing the behavior			**+**	
5.6	Information about emotional consequences	Provide information (e.g. written, verbal, visual) about emotional consequences of performing the behavior		**–**	**+**	
6.1	Demonstration of the behavior	Provide an observable sample of the performance of the behavior, directly in person or indirectly e.g. via film, pictures, for the person to aspire to or imitate			**+**	**+**
6.2	Social comparison	Draw attention to others’ performance to allow comparison with the person’s own performance	**+**			
6.3	Information about others’ approval	Provide information about what other people think about the behavior. The information clarifies whether others will like, approve or disapprove of what the person is doing or will do			**+**	
7.1	Prompt/cues	Introduce or define environmental or social stimulus with the purpose of prompting or cueing the behavior	**+**	**mixed**		
15.3	Focus on past success	Advise to think about or list previous successes in performing the behavior	**+**			

Improvement in overall diet includes outcomes such as fruits and vegetables together with reduced fat intake and caloric intake. "+" indicates that interventions using the specific BCT are more effective at changing the behavioral outcomes compared to interventions that do not integrate the specific BCT; "mixed" indicates mixed findings about the effectiveness of that behavior change technique on those outcomes (including positive and negative effects); "-" indicates that interventions using the specific BCT are less effective at changing the behavioral outcomes compared to interventions that do not integrate the specific BCT. * SSB refers to Sugar-Sweetened Beverages. Boxes are left blank or unfilled when no data were available.

This meta-synthesis of the literature offers a list of BCTs that can positively contribute to successful eating behavior changes and thus serve as a basis to design individual behavior change interventions based on available empirical evidence. Although this question should be explored further, it appears that, depending on our goal (promote or hinder the consumption of certain food groups), different BCTs should be implemented. In other words, some BCTs could be more effective for targeting a reduction in consumption (e.g., reduce meat consumption) than for targeting an increment (e.g., promote fruits and vegetables intake) [25]. It is also worth mentioning that different eating behaviors probably do not have the same behavioral plasticity or, in other words, are not equally easy to change and sustain [64]. It is likely that some deeply established behaviors, such as meat consumption, are more difficult to change and require greater emphasis when designing an intervention than other behaviors, such as increased fruit and vegetable consumption [46]. Beyond the type of the BCTs to be applied, it seems that using several BCTs in the same intervention would lead to more effective results [53], up to a certain threshold where the manipulation of too many interventional components can have detrimental effect [65]. The most effective combination of BCTs and the order in which they have to be implemented is something that requires further research [66]. Additionally, the effectiveness of eating behavior interventions may depend on the characteristics and motivations of targeted individuals. The assessment of intervention features that are most likely to successfully tackle eating behaviors in those with less sustainable diets is definitely needed.

## 4 Limitations of previous individual behavior change interventions and perspectives

Past eating behavior interventions mainly focused on specific food groups, such as fruit and vegetable intake, reducting meat consumption, or reducing fat and energy intake, instead of taking a global dietary approach. Focusing on single food groups comes with a particular limitation: the lack of consideration of potential behavioral spillovers (also called rebound effects when expressed in terms of energy). Spillovers occur when a change in one specific behavior leads to secondary order changes in other related behaviors [67]. In some cases, these spillovers can be positive, when one favorable change to one’s diet leads to another favorable behavioral change; but they can also be negative, when one positive change comes with a secondary order, likely unplanned, change in another behavior, compromising the overall effect of the intervention. These negative spillovers may occur *among food groups*, for instance when an increment in vegetable consumption “licenses” a subsequent increase in added sugars, offsetting the health benefits of increased vegetable intake [68], or when meat reduction, such as pork or poultry, is compensated by an increase in cheese (one of the food products with a high environmental impact, not only by weight, but also by protein and energy content), incrementing the dietary environmental impact [69]. Spillovers also can arise in behaviors unrelated to food choices. These can occur within the *whole food domain* (e.g., the environmental benefits of adopting a low environmental impact diet would be offset if followed by more food wasted) [70]; between *high-environmental impact behaviors* (e.g., when the money saved from reducing meat consumption is re-directed to goods or services with high environmental impact, for instance, traveling by plane) [71]; or *between health-related behaviors* (e.g.*,* when the adoption of a healthier diet could license the lack of practice of a regular physical activity) [72].

Additionally, it is quite rare to find literature on eating behavior change that simultaneously aims at promoting diets that are both healthier and more sustainable. While the healthiness and environmental impact of foods/diets usually go hand in hand, this is not always the case. For example, fish is a healthy food, but - in some instances - its environmental impact is notorious [73]. Similarly, the promotion of healthy diets may lead to health benefits, but at the expenses of a higher dietary environmental impact depending on the foods added and removed [74,75]. Even healthy foods with low environmental impact, if obtained from unfair sources, could compromise the wellbeing and even food security of producers and other stakeholders all along the food system [76]. All these dimensions should be considered together to achieve the maximum co-benefits, avoiding unintended spillovers and trade-offs among domains.

We argue here that addressing spillovers and trade-offs is crucial when designing interventions to promote sustainable healthy diets to ensure an overall positive impact of the intervention in terms of human health, planetary health and social equity. Based on this, and considering the major dietary changes required in high-income countries for the general adoption of sustainable healthy diets [25], we propose an actionable definition based on previous guiding principles [18], but explicitly tailored to researchers, practitioners and policy-makers aimed at the promotion of sustainable healthy diets:

*When promoting sustainable healthy diets, the consumption of animal-sourced proteins, such as meats, especially red and processed meat, and dairies should be reduced, and substituted by plant-based proteins,* i.e. *legumes and nuts. At the same time, the consumption of whole grains should be emphasized over the refined versions, unsaturated and unrefined oils (*e.g.*, virgin olive oil, canola oil) should be promoted over other dietary fats (*e.g. *butter, coconut oil), and the consumption of water should be targeted as a main dietary beverage, over sweetened beverages and alcoholic drinks. The consumption of vegetables and fruits should be incentivi*z*ed, while the intake of foods rich in sugars, salt and/or unhealthy fats should be decreased [**25**]. All these behavioral changes should be monitored accounting for potential negative spillovers across food groups, making sure that all required nutrients and energy are consumed, neither in deficiency nor in excess. Special attention should be paid also to spillovers across high-environmental impact and health-related behaviors, and to potential trade-offs within the dimensions of dietary sustainability: human health, environmental impact and socio-economic wellbeing.*

Additionally, and as pointed out elsewhere [64], future behavior change interventions targeting eating will also have to overcome specific limitations of previous interventions related to pro-environmental and/or health behavior change. This includes *i*) the adoption of study designs allowing for strong causal inferences such as randomized control trials but also N-of-1 trials, offering higher possibilities of tailoring and continuous optimization, at lower financial costs [77]; *ii*) longer periods of monitoring, such as several months, to capture accurate patterns of change in eating behaviors over time, and at the right resolution (e.g., changes happening from week to week) [78]; and *iii*) the inclusion of citizen and directly targeted users in the development process of such interventions to increase the chances for its acceptability and feasibility, and to gather relevant information on people's needs [79]. (See [80] for a recent initiative meeting some of these criteria).

Further, the development of reliable measurement tools and scoring procedures to both briefly screen (see [81] for a relevant dietary screener for assessing sustainable diet) as well as continuously monitor individual's changes in sustainable diets (as the tool developed for this intervention [80]), together with the aforementioned spillovers, is definitely necessary. Traditional dietary indices have only focused on the dietary healthiness and nutrient adequacy, leaving aside the environmental, socio-cultural and economic dimensions of food; the scoring criteria of those few that considered all those dimensions at once was not easily applied by consumers, nutritional practitioners or behavioral researchers [82,83]. Some efforts have been undertaken in recent years for the development of more practical scores for the assessment of sustainable healthy diets, but they still lack consideration of the socio-cultural and economic dimensions, and other behaviors beyond diet [80,84,85]. The consideration of these factors is key to evaluating the overall effect of the intervention on sustainability as a whole. The utilization of more objective data collection, in parallel with self-reported behavioral outcomes, such as food photos, could also help improving the evaluation of these interventions [86].

## 5 Conclusions

A social transformation toward a lifestyle in general, and diet in particular, that fit within planetary boundaries is urgent. Beyond structural changes, individual behavioral change is deeply needed in order to accelerate such a transition, notably among high-income groups within and between countries. The present viewpoint offers a starting point for the design of future individual interventions for sustainable healthy diets based on available evidence in terms of effective behavior change techniques for promoting different eating behaviors. We also draw important new perspectives related to the consideration of various spillovers and trade-offs when changing one’s behavior. Notably, based on this review, we argue that more interventional studies should adopt a holistic conception of sustainable diets, rather than focusing solely on specific food groups such as red meat. Similarly, when interventions are designed to target specific food groups, we recommend that potential rebound effects be measured, or ideally, anticipated and addressed within the intervention design. Ultimately, this viewpoint is also a call for more multidisciplinary collaborations between environmental, socio-economic, nutrition and behavioral researchers to develop future relevant interventions promoting sustainable healthy diets in their full complexity.

## CRediT authorship contribution statement

**Ujué Fresán:** Writing – original draft, Conceptualization. **Paquito Bernard:** Writing – review & editing, Conceptualization. **Vera Araújo-Soares:** Writing – review & editing, Conceptualization. **Simon J Lloyd:** Writing – review & editing, Conceptualization. **Guillaume Chevance:** Writing – review & editing, Writing – original draft, Conceptualization.

## Declaration of competing interest

The authors declare that they have no known competing financial interests or personal relationships that could have appeared to influence the work reported in this paper.
